# Serotonin transporter gene polymorphism (5-HTTLPR) L allele interacts with stress to increase anxiety symptoms in Chinese adolescents: a multiwave longitudinal study

**DOI:** 10.1186/s12888-015-0639-y

**Published:** 2015-10-14

**Authors:** Qingsen Ming, Yun Zhang, Jinyao Yi, Xiang Wang, Xiongzhao Zhu, Shuqiao Yao

**Affiliations:** Medical Psychological Institute, The Second Xiangya Hospital, Central South University, NO.139 Middle Renmin Road, Changsha, Hunan 410011 China; National Technology Institute of Psychiatry, Key Laboratory of Psychiatry and Mental Health of Hunan Province, Changsha, Hunan 410011 China

**Keywords:** Gene-environment, Stress, Anxiety, Adolescent

## Abstract

**Background:**

Previous studies of the interaction between a functional polymorphism in the serotonin transporter gene-linked promoter region (5-HTTLPR) and stress in anxiety-related phenotypes have produced inconsistent results. The aim of the current study was to examine the effect of the 5-HTTLPR × stress interaction on anxiety symptoms in Chinese adolescents.

**Methods:**

A total of 651 healthy adolescents [323 females and 328 males; age 14–17 (mean = 16.27, standard deviation = 0.77)] participated in this study. At the initial assessment, participants completed self-report measures assessing anxiety symptoms, depressive symptoms and stressful life events. Additionally, anxiety symptoms and stressful life events were assessed once every 3 months for the subsequent 9 months. A hierarchical linear model (HLM) was used to investigate the 5-HTTLPR × stress interaction.

**Results:**

The HLM indicated no main effect of 5-HTTLPR on anxiety symptoms. Significant 5-HTTLPR × stress interaction effect in predicting anxiety symptoms was found. Specifically, individuals with the 5-HTTLPR L allele exhibited more anxiety symptoms related to stressful life events.

**Conclusions:**

The association between stress and anxiety symptoms is moderated by 5-HTTLPR. The 5-HTTLPR L allele increases individuals’ vulnerability to anxiety under stress situations.

## Background

Anxiety disorders are complex mental disorders that place an enormous burden on individuals and society [1]. Environmental stress, often indexed by stressful life events (SLEs), has been consistently linked to anxiety. Nevertheless, the effects of SLEs on anxiety differ among individuals, and not all people who experience SLEs develop anxiety disorders [2], suggesting the existence of variation in individuals’ susceptibility to stress.

Susceptibility to stress may have biological roots, especially in the serotonergic system. Serotonin [5-hydroxytryptamine (5-HT)] is an important monoamine neurotransmitter involved in the regulation of several psychological activities, such as emotion, cognition, circadian and neuroendocrine rhythms (food intake, sleep and sexual activity) [3]. Among genes of the 5-HT system, the serotonin transporter (5-HTT) gene has captured particular attention because 5-HTT is involved in the reuptake of 5-HT at brain synapses. The 5-HTT gene has several polymorphisms, including a functional polymorphism consisting of a 44–base pair insertion/deletion in the 5′ promoter region, known as the serotonin transporter–linked promoter region polymorphism (5-HTTLPR). 5-HTTLPR includes long (L) and short (S) alleles that influence the rate of 5-HT transcription. Specifically, the S allele has lower transcriptional efficiency compared with the L allele [4].

Focusing on 5-HTT, Lesch et al. [4] first linked anxiety-related traits with the S allele of 5-HTTLPR. However, this association was observed inconsistently in subsequent studies of anxiety-related phenotypes and was difficult to replicate [5–7]. A possible explanation for the mixed results is that genetic vulnerability might manifest in subjective markers of anxiety, but only when activated by environmental factors, such as stress. In recent years, researchers have paid attention to the interplay between genetic and environmental (G × E) factors in anxiety. Several studies have investigated the 5-HTTLPR × stress interaction in anxiety-related phenotypes. Unlike risk role of 5-HTTLPR S allele in most depression studies [8], there was no consolidated conclusion about 5-HTTLPR × stress interaction in anxiety to date. Gunthert et al. [9] and Petersen et al. [10] observed that individuals with at least one S allele who experienced more stressors reported more anxious mood. Conversely, Grabe et al. [11] found an interactive effect of the higher active L allele and traumatic events on anxiety disorder. However, a few studies indicated no interaction effect between 5-HTTLPR and SLEs on anxious/depressed symptoms or anxiety sensitivity [12, 13]. These conflicting results for the 5-HTTLPR × stress interaction may be due to differences in sample age, ethnicity (e.g., 5-HTTLPR allele frequency varies considerably according to ethnic background), as well as the methods used to rate predictors and outcomes.

Beyond the inconsistent findings, previous studies have several major limitations. Most studies conducted to test G × E factor interaction in anxiety used cross-sectional designs. Without longitudinal measures of predictors and outcomes, researchers cannot determine whether this interaction predicts the development of anxiety over time. Furthermore, the majority of previous studies focused predominantly on adult samples, few studies have thoroughly examine these effects and interactions in adolescence, one of the most important periods for the onset of anxiety. Adolescence is marked by major changes in physiology, interpersonal relationships and social interests. Because of unique stressors related to school work and socialization, anxiety symptoms and the prevalence of anxiety disorders increase substantially in adolescence [14]. Thus, adolescence is a crucial period to study the pathogenesis of genetic and environmental factors’ effects on anxiety. Moreover, few studies have considered the effects of depressive symptoms on anxiety symptoms. As depression and anxiety are closely related in children and adolescents [15], it is necessary to control for depressive symptoms when testing G × E interaction in anxiety. In addition, 5-HTTLPR × stress studies have yielded inconsistent results in different ethnicities [16, 17]. Due to ethnical differences, it is necessary to test whether the conclusions of 5-HTTLPR × E studies among other ethnicities may apply to Chinese populations.

To the best of our knowledge, no study has investigated how 5-HTTLPR and SLEs may interact to predict changes in anxiety symptoms over time among Chinese adolescents. Increases in anxiety symptoms over time are expected to be related to both changing levels of stress and individuals’ genetic predispositions. We have reported interaction effects of 5-HTTLPR × stress on depression in Chinese adolescent girls in previous study [18]. In the present study, we thus used the same longitudinal design to investigate whether the interaction between 5-HTTLPR and SLEs can predict anxiety symptoms in Chinese adolescents.

## Methods

### Participants

All subjects were volunteers recruited from two public senior high schools in Hunan province of China by posters and media advertisements. Participants with neurological diseases, and/or past or current episodes of anxiety disorders, major depression disorder, manic disorder, bipolar disorder, schizoaffective disorder or schizophrenia were excluded. Of the 692 adolescents administered the clinical interviews, 41 were excluded for meeting the criteria for lifetime major depressive disorder (9, 1.3 %), generalized anxiety disorder (11, 1.6 %), compulsive disorder (16, 2.3 %) or specific phobia (5, 0.7 %). Finally, the study sample consisted of 651 healthy students (323 females and 328 males) aged 14–17 (mean = 16.27, standard deviation = 0.77). All subjects were of Han ancestry, the predominant ethnic group in China.

### Procedure

This study complied with the Code of Ethics of the World Medical Association and was approved by the Ethics Committee of Second Xiangya Hospital, Central South University. All participants and their parents received detailed information and provided written informed consents. Trained researchers who were graduate students at Second Xiangya Hospital performed clinical assessments and administered questionnaires. Neurological physical examination and the clinical interviews were conducted one-on-one with each participant outside of class time. Interviews consisted of two parts. The Anxiety Disorders Interview Schedule for Children for DSM-IV was used to assess anxiety disorders [19]. Affective disorder, schizoaffective disorder and schizophrenia were diagnosed using the Schedule for Affective disorder and Schizophrenia for School-Age Children [20]. At initial assessment, each subject completed the Chinese version of Multidimensional Anxiety Scale for Children (MASC-C) [21], Center for Epidemiological Studies Depression Scale (CES-D) [22], and Adolescent Life Events Questionnaire (ALEQ) [23]. In addition, an oral swab was collected from each participant for 5-HTTLPR genotyping during the initial assessment. The researchers returned to the schools to conduct assessments every 3 months for the subsequent 9 months (i.e., at 3, 6, 9 months). Participants completed the MASC-C and ALEQ at each follow-up assessment.

### Measurements

#### Multidimensional anxiety scale for children

The MASC-C is a 39-item measure that assesses the severity of anxious symptoms in the past week [21]. Each item consists of a statement that youth rate on a four-point Likert scale ranging from 0 (never applies to me) to 3 (often applies to me). Total scores range from 0 to 117, with higher scores indicating higher levels of anxiety symptoms. The MASC-C has been demonstrated to be reliable and valid [21]. In the current study, Cronbach’s alpha values for the scale ranged from 0.90 to 0.95 across administrations, indicating strong internal consistency.

#### Center for epidemiological studies depression scale

The CES-D is a 20-item measure designed to assess the current level of depressive symptoms, with emphasis on the affective component, and depressive mood in general populations [22]. Each item consists of one symptom. Participants rated the frequency of each symptom within the past week on a four-point scale (0, <1 day; 1, 1–2 days; 2, 3–4 days; 3, 5–7days). Total scores range from 0 to 60, with higher scores indicating higher elevations in depressive symptoms. The Chinese version of the CES-D has shown high degrees of reliability and validity [24]. The Cronbach’s alpha value for the scale was 0.89 at the initial assessment, indicating high internal consistency.

#### Adolescent life events questionnaire

The ALEQ is a self-reported questionnaire developed by Hankin and Abramson [23] to assess a broad range of negative life events (e.g., school/achievement problems, friendship and romantic problems, and family problems) that occur during adolescence. Participants rated the frequency of negative life events within the past month on a five-point scale (1, never; 2, rarely; 3, sometimes; 4, usually; 5, always). Total scores range from 70 to 350, with higher scores reflecting a greater number of negative life events. Past research has found the ALEQ to be reliable and valid when used in Chinese adolescents [25]. In the current study, Cronbach’s alpha values for this scale ranged from 0.95 to 0.97 across administrations, indicating high internal consistency.

### 5-HTTLPR genotyping

Genomic DNA was extracted from exfoliated buccal cells using the TIANamp Swab DNA Kit (TIANGEN Biotech, Beijing, China) according to standard procedures. 5-HTTLPR genotyping was performed using the primers described by Heils et al. [3] (forward: 5′-GGCGTTGCCGCTCTGAATTGC-3′; reverse: 5′-GAGGGACTGAGCTGGACAACCCAC-3′). Polymerase chain reaction (PCR) amplification was conducted using a Perkin-Elmer GeneAmp PCR System 2400 (Applied Biosystems, USA). Amplification system was performed in a volume of 25 μL containing 50ngDNA template, 9.5 μL nuclease-free water, 0.4 μM each primer, 0.2 mM dNTPs, 10 mM Tris–HCl (pH = 8.3), 50 mM KCl, 1.5 mM MgCl_2_, and 1U GoTaq DNA polymerase (Promega, USA). The cycling conditions were: (1) initial denaturation at 94 °C for 3 min, (2) 35 cycles of amplification (denaturation at 95 °C for 30s, annealing at 62 °C for 30s, and synthesis at 72 °C for 45 s), and (3) final extension at 72 °C for 7 min. The amplification products were resolved on a 1.5 % agarose gel by electrophoresis and visualized by Du Red staining (Biosharp, USA). Fragment sizes were determined on a Bio-Rad Gel Doc XR+ system (Bio-Rad, USA) by comparison with molecular length standards (50 bp ladder, TIANGEN Biotech). The results were fully validated in approximate 5 % of genotyped individuals who were randomly selected for retesting through the same procedure.

### Statistical analysis

A hierarchical linear model (HLM) was used to investigate whether interaction between 5-HTTLPR and stress can predict the level of anxiety symptoms. Analyses were carried out using the SAS (version 9.0, SAS Institute) MIXED procedure and maximum likelihood estimation. A two-level structure of model was handled. Level 1 was 4-time follow-up measurements within each subject (fluctuation in anxiety and stress over times within each subject), and level 2 was all subjects (different 5-HTTLPR genotypes between subjects). The dependent variable was within-subject fluctuation in MASC-C scores during the follow-up period (ANXIETY). The primary predictors of ANXIETY were 5-HTTLPR and fluctuations in ALEQ scores during the follow-up period (STRESS). As STRESS was a within-subject predictor, ALEQ scores were centered at each individual’s mean prior to analyses, such that STRESS reflected the upward or downward fluctuation in an individual’s level of stress compared with his/her mean level of stress. 5-HTTLPR was treated as a three-classification variable by triallelic genotyping (LL = −1, SL = 0, SS = 1). To control for individual differences in age, gender, and baseline anxiety symptoms, these variables were included in this model. CES-D scores obtained at timepoint 1 were also included in the model to control for depressive symptoms. The two-level model for subject *i* at timepoint *t* was:

- Level 1 (within-subject)
- ANXIETY_*ti*_ = β_0*i*_ + β_1*i*_(STRESS)_*ti*_ + e_*ti*_
- Level 2 (between-subject)
- β_0*i*_ = γ_00_ + γ_01_(timepoint 1 MASC-C)_*i*_ + γ_02_(timepoint 1 CES-D)_*i*_ + γ_03_(Age)_*i*_ + γ_04_(Gender)_*i*_ + γ_05_(5-HTTLPR)_*i*_ + u_0*i*_
- β_1*i*_ = γ_10_ + γ_11_(5-HTTLPR)_*i*_ + u_1*i*_.
- The mixed model:
- ANXIETY_*ti*_ = γ_00_ + γ_10_(STRESS)_*ti*_ + γ_05_(5-HTTLPR)_*i*_ + γ_01_(timepoint 1 MASC-C)_*i*_ + γ_02_(timepoint 1 CES-D)_*i*_ + γ_03_(Age)_*i*_ + γ_04_(Gender)_*i*_ + γ_11_(STRESS)_*ti*_(5-HTTLPR)_*i*_ + [u_0*i*_ + u_1*i*_(STRESS)_*ti*_ + e_*ti*_]

## Results

### Frequency of 5-HTTLPR genotypes

The frequency distributions of the 5-HTTLPR genotypes are as following: SS: 351 (173 boys, 178 girls), SL: 252 (130 boys, 122 girls), LL: 48 (25 boys, 23 girls). The genotype frequencies were consistent with Hardy-Weinberg equilibrium (*χ*^*2*^ = 0.034, *p* >0.05). No gender difference in 5-HTTLPR frequency distributions was observed.

### Descriptive anxiety, depression and stress data

One sample Kolmogorov-Smirnov tests showed that MASC-C scores in 4 measurements all accorded with normal distribution (all *p* >0.05). The Pearson’s correlation coefficients between CES-D scores and MASC-C scores in 4 measurements ranged from 0.36 to 0.58 (all *p* <0.001). The means and standard deviations of all assessments and their gender differences are presented in Table 1. Overall, MASC-C and ALEQ scores decreased. Females reported higher levels of anxiety symptoms than did males at all assessments. Gender differences in MASC-C scores were significant at the initial assessment (t = −2.34, *p* <0.05) and at 6 months (t = −2.24, *p* <0.05). Males reported more SLEs than did females at 9 months (t = 2.87, *p* <0.05). Differences of scores in all assessments between 5-HTTLRP genotypes are presented in Table 2. There were significant differences between 3 genotypes on ALEQ scores at 9 months (F = 3.41, *p* <0.05).

**Table 1:** Means and standard deviations for all assessments and their gender differences

	Total	Gender differences			
		Females	Males	*t*	*p*
CES-D (Timepoint 1)	11.72(8.65)	11.25(8.78)	12.21(8.53)	0.85	0.40
MASC-C					
Timepoint 1	44.72(16.17)	47.09(16.12)	42.24(15.79)	−2.34	0.02
At 3 months	34.21(17.99)	36.39(18.17)	31.96(17.61)	−1.88	0.06
At 6 months	32.20(18.55)	34.69(18.86)	29.14(17.78)	−2.24	0.03
At 9 months	29.61(18.60)	31.52(18.39)	27.60(18.68)	−1.62	0.11
ALEQ					
Timepoint 1	137.39(36.35)	133.43(33.28)	141.80(39.24)	1.49	0.14
At 3 months	113.15(33.55)	115.06(30.66)	111.14(36.36)	−0.92	0.36
At 6 months	112.61(33.78)	113.38(32.05)	111.74(35.77)	−0.36	0.72
At 9 months	108.11(33.18)	103.81(24.89)	113.03(40.21)	2.87	0.04

**Table 2:** Differences of scores in all assessments between 5-HTTLPR genotypes

	SS	SL	LL	*F*	*p*
CES-D (Timepoint 1)	11.85(8.65)	11.46(8.84)	12.11(11.54)	0.31	0.74
MASC-C					
Timepoint 1	44.43(15.77)	44.85(15.80)	46.12(18.68)	1.82	0.16
At 3 months	33.89(15.93)	34.37(16.93)	35.74(18.90)	2.49	0.09
At 6 months	32.09(17.74)	32.18(18.25)	33.13(18.94)	0.89	0.41
At 9 months	29.29(17.60)	29.91(18.80)	30.36(21.90)	0.90	0.41
ALEQ					
Timepoint 1	137.61(39.57)	137.23(33.42)	136.62(45.86)	0.07	0.93
At 3 months	114.01(36.93)	113.16(33.09)	106.81(38.35)	1.66	0.19
At 6 months	113.93(35.45)	112.17(33.02)	105.26(43.00)	1.81	0.17
At 9 months	109.68(38.66)	107.93(28.71)	97.57 (40.21)	3.41	0.03

### Statistical analyses of interaction between 5-HTTLPR and stress

**Fig. 1:**
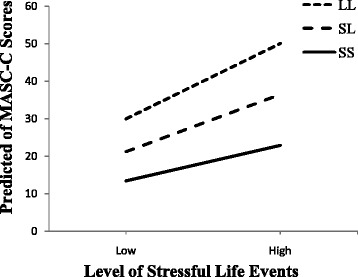
The predicted slope between stress and anxiety symptom for different genotypes. The high/low levels of stressful life events meant plus/minus 1.5 × mean within-subject standard deviation. Although increases in stressful life events were all associated with increases in anxiety symptoms for individuals with different genotypes, LL carriers have a moderately heightened anxiety response to stress compared to SS carriers

To select a covariance structure for our analyses, we fitted the models utilizing each structure and chose the best fit based on Akaike information criterion (AIC and AICC) and Schwarz Bayesian criterion (BIC). The best fit was a heterogeneous autoregressive structure (ARH[1]). With respect to random effects, the ARH[1] parameter (*p* <0.001), random slope (*p* <0.01) and random intercept (*p* <0.001) were all retained in the model. Table 3 showed the estimates of covariance parameter for the final model. Preliminary analyses indicated no gender differences in models with 5-HTTLPR × stress interaction, and thus fixed-effects component of the model analyses are presented for the sample as a whole. Analyses of HLM showed a significant main effect of stress on anxiety symptoms (B = 0.21, *p* <0.001, Table 4). No significant main effect of 5-HTTLPR on anxiety symptoms was found (B = 0.32, *p* >0.05). After controlling for age, gender, initial anxiety and depressive symptoms, a significant two-way, cross-level interaction between 5-HTTLPR and stress were detected (B = −0.08, *p* <0.01). As stress levels increase, the anxiety level among SS carriers appears to increase at a slower rate compared to LL carriers. To present the form of this interaction, the model summarized in Table 4 was used to calculate predicted anxiety symptom scores for participants with SS, SL and LL genotypes who experienced a low or high level of stressful life events (plus or minus 1.5 × mean within-subject standard deviation) without controlling for age, gender or depressive symptoms (see Fig. 1).

**Table 3:** Estimates for covariance parameters of the final model

Cov Parm	Estimate	SE	Z	*p*
UN (1, 1)	212.07	15.69	13.52	<0.001
UN (2, 2)	0.02	0.01	3.06	<0.01
ARH[1]	5.87	0.31	19.24	<0.001
Residual	134.33	6.98	19.24	<0.001

**Table 4:** Estimation of stress, 5-HTTLPR and 5-HTTLPR × Stress predicting anxiety symptoms

Predictors	B	SE	*df*	*t*	*p*
Baseline anxiety symptoms	10.00	0.59	1298	17.11	<0.001
Baseline depressive symptoms	0.30	0.07	1298	4.39	<0.001
Age	1.01	0.69	647	1.46	0.14
Gender	3.68	0.99	647	3.73	<0.001
Stress	0.21	0.02	1298	10.48	<0.001
5-HTTLPR	0.32	0.75	1298	0.42	0.68
5-HTTLPR × Stress	−0.08	0.02	1298	−3.04	<0.01

Note: Baseline Anxiety Symptoms as assessed by Timepoint 1 MASC-C, Baseline Depressive Symptoms as assessed by Timepoint 1 CES-D, Stress as assessed by with-in subject fluctuations in ALEQ scores during the follow-up intervals

## Discussion

As found in many other studies [26–28], females reported higher levels of anxiety symptoms than did males at each assessment in the current study, indicating that females are more likely to experience anxiety symptoms in adolescence. Stress and anxiety symptom levels were highest at the initial assessment and decreased during the follow-up period. A possible explanation for this phenomenon is that the students had just entered senior high school when the first assessment was carried out. This major transition may have involved increased competition and academic pressure, elevating stress and anxiety symptoms. The decreases in stress and anxiety levels may reflect acclimation to senior high school.

No evidence of a 5-HTTLPR main effect was found in the present study, in accordance with the findings of previous researches on the association between 5-HTTLPR and anxiety symptoms [6], generalized anxiety disorder [7] or anxiety sensitivity [13], suggesting that 5-HTTLPR does not directly affect anxiety symptoms.

Regarding gene-environment interaction, this is the first longitudinal study to clarify the interaction between 5-HTTLPR and stress in the prediction of anxiety symptoms in Chinese populations. In consideration of the limitation of sample size in our previous depression study [18], along with high correlation between depression and anxiety in adolescents [15], we performed the current study in a larger sample and controlled for the effects of depressive symptoms on fluctuation of anxiety symptoms to improve reliability of the results. A two-way interaction of 5-HTTLPR and SLEs was detected in the current study, LL genotypes increase genetic vulnerability to the experience of anxiety symptoms slightly in response to daily stressors. Consistent with our finding, an interaction effect of the 5-HTTLPR LL genotypes and family adversity [29], traumatic events [11], or childhood maltreatment [30], respectively, has been observed for anxiety disorders or anxiety sensitivity. In contrast, some studies indicated significant interactions between S allele and SLEs or childhood maltreatment on anxiety-related phenotypes [9, 10, 31]. Other studies even suggested no interaction of 5-HTTLPR × stress on anxiety [12, 13].

Several reasons may account for inconsistencies in 5-HTTLPR-related association findings in anxiety. Firstly, ethnic background may be one of the major reasons for the inconsistent results. For instance, most 5-HTTLPR × E studies in depression point to a general tendency for detrimental effect of the 5-HTTLPR S allele. But one of the few study in Chinese population indicated that individuals carrying L alleles could be susceptible to major depression when exposed to negative life events [17]. There were essential uncertainties in ethnic differences of 5-HTTLPR allele frequency, 5-HTT availabilities, 5-HTT uptake and the central serotonergic activity, which might be responsible for the inconsistency. Frequency of the L allele is much lower than that of S allele in Chinese population [32] and Japan population [33] but higher in Caucasians or African-Americans [4, 34]. Meta-analyses have demonstrated the different effects of 5-HTTLPR on selective serotonin reuptake inhibitors (SSRIs) efficacy between Caucasians and Asians [35, 36]. Moreover, functional magnetic resonance imaging (fMRI) studies have shown a link between S allele and higher amygdala activation in response to emotional stimuli in Caucasians [37–39], whereas in Asians L allele was associated with amygdala hyperactivation [40]. Therefore, these fascinating questions of different 5-HTTLPR effects between Caucasian and Asian populations require further investigation. It was notable that results of the current study were inconsistent with our previous depression study which indicated risk effect of S allele in girls [18]. The reasons were various (e.g. sample size, other gene variants, and different pathological mechanisms of 5-HTTLPR in anxiety and depression) and should be explored in future.

Second, sample age spanning may be another possible reason contributing to inconsistencies. The majority of studies using subjects whose ages spanned a wide range might neglect developmental issues. Given the dynamics of genetic influences across the lifespan, the impact of genetic factors is likely to depend on developmental stages [41]. Indeed, findings on the neurobiological function of the 5-HTTLPR were not stable between adolescents and adults. A fMRI study assessing amygdala function demonstrated that adolescents with current anxiety or major depressive disorder who were carries of 5-HTTLPR LL genotype exhibited higher amygdale activation to fear faces [42]. This finding was contrary to those reported from affected adults, indicating greater amygdala response in S allele carries [43].

Furthermore, other genetic variants are variables to be considered in light of the inconsistent findings. It is conceivable that a single gene variation and a certain environmental factor cannot completely reflect the complexity of gene-environment interactions. Thus, the exploration of multiple genetic variations interacting with multiple environmental factors may be needed. Regarding further genetic factors, there are already studies reporting interactions between 5-HTTLPR, other polymorphisms and stress on depression [17, 44]. Therefore, future research should consider gene-gene-environment interactions to gain a more comprehensive understanding of the pathological mechanism of anxiety.

Strengths of the present study included its longitudinal design, which allowed us to control for pre-existing anxiety and depression symptoms to ascertain the effects of stress and 5-HTTLPR on fluctuation of anxiety symptoms in follow-up intervals. We examined 5-HTTLPR, in conjunction with within-subject fluctuations in stress level, to predict within-subject fluctuations in the level of anxiety symptoms. This idiographic approach and relatively reliable estimate of each adolescent’s degree of stress reaction minimized the effects of individual differences in variables. The study was also strengthened by a sample of homogenous population that reduced biases.

However, several limitations of the current study should be noticed. First, self-report measures were used to assess SLEs, which may be less accurate than the use of contextual stress interviews. Further research would benefit from using contextual stress interviews in a multiwave framework. Second, the present study examined the G × E interaction only in adolescents aged 14–17 years. Additional research is needed to test whether findings from this study can be generalized to younger adolescents or children. Third, the sample of this study was non-clinical sample which limited the generalizability of the findings to patients with anxiety disorders. Additionally, the effect size of 5-HTTLPR × stress interaction was modest. It is important to replicate these results in future so that we can be more confident in this interaction. Finally, a single nucleotide polymorphism (SNP) rs25531 within L allele has been described [45]. Due to this SNP, the L allele can be further categorized into L_A_ and L_G_ allele, with L_G_ allele functionally equivalent to S allele [16]. In the current study, genotype analysis did not differentiate between L_A_ and L_G_ alleles. However, considering the frequency of L allele frequency in the present study (0.267) and the minor allele frequency of rs25531 [G = 0.138, according to the NCBI Variation Database (http://www.ncbi.nlm.nih.gov/SNP/)], the L_G_ allele should occur infrequently in our study sample. Moreover, present findings remain unclear in studies reclassified L_G_ allele with lower transcription efficiency. And a study by Martin et al. [46] questioned the functional interpretation of the L_G_ allele.

## Conclusions

In conclusion, results of the current study indicate an interaction between 5-HTTLPR and stress in the prediction of anxiety symptoms among Chinese adolescents. Individuals with the L allele exhibited moderately heightened anxiety response to stress. If the results can be replicated, the current study will provide new evidence for exploring the roles of genetic and environmental factors in the pathological mechanism of anxiety.
